# Extracellular vesicles in malaria: an agglomeration of two decades of research

**DOI:** 10.1186/s12936-021-03969-8

**Published:** 2021-11-20

**Authors:** Tosin Opadokun, Petra Rohrbach

**Affiliations:** Institute of Parasitology, McGill University, Montreal, Canada

**Keywords:** Extracellular vesicles, Exosomes, Microvesicles, *Plasmodium*, Malaria, Biomarkers, Pathogenesis

## Abstract

Malaria is a complex parasitic disease, caused by *Plasmodium* spp. More than a century after the discovery of malaria parasites, this disease continues to pose a global public health problem and the pathogenesis of the severe forms of malaria remains incompletely understood. Extracellular vesicles (EVs), including exosomes and microvesicles, have been increasingly researched in the field of malaria in a bid to fill these knowledge gaps. EVs released from *Plasmodium*-infected red blood cells and other host cells during malaria infection are now believed to play key roles in disease pathogenesis and are suggested as vital components of the biology of *Plasmodium* spp. Malaria-derived EVs have been identified as potential disease biomarkers and therapeutic tools. In this review, key findings of malaria EV studies over the last 20 years are summarized and critically analysed. Outstanding areas of research into EV biology are identified. Unexplored EV research foci for the future that will contribute to consolidating the potential for EVs as agents in malaria prevention and control are proposed.

## Background

Malaria is one of the oldest diseases known to man [1] yet it remains a global public health problem with over 3.4 billion of the world’s population at risk of infection [2]. It is a complex parasitic disease and infection may progress from asymptomatic malaria through uncomplicated, severe, and fatal malaria, with non-immune children and adults at greater risk of severe malaria and death. The foremost factor determining the clinical manifestation is the infecting parasite species. Severe malaria is known to occur by infection with the human-only *Plasmodium falciparum* and *Plasmodium vivax* species, as well as by zoonotic *Plasmodium knowlesi* infections [3]. Major severe malaria syndromes include severe anaemia, metabolic acidosis, multiorgan failure and cerebral malaria (CM), which is characterized by coma and other neurological complications [4]. CM remains unreported in knowlesi infections [5, 6], but is being increasingly reported in children and adults infected with *P. vivax* [7–11]. *Plasmodium falciparum* is the most medically important *Plasmodium* spp. and is known to be highly associated with CM [4, 12]. CM is the leading cause of malaria-related deaths and in 2018, *P. falciparum* accounted for over 90% of the globally estimated 400,000 malaria deaths [2].

The pathogenic basis of CM is poorly understood but involves profound adhesive properties of *P. falciparum* and host immune responses that converge on a compromised brain microvasculature [4, 13–15]. CM due to *P. vivax* is a fairly new concept and remarkably less is known about the pathogenesis of the severe forms of vivax malaria [16]. Towards elucidating the pathophysiology of severe malaria, particularly CM, extracellular vesicles (EVs) as mediators of long-distance cell-cell communication, have been proposed as integral components in the pathobiology of malaria [17–19].

Since the earliest observations of EVs in human plasma were made [20, 21], their importance in numerous physiological and pathological processes has become increasingly evident as decades of intense research on EVs released from mammalian cells ensued [22–24]. Recently, investigation of EVs in pathogenic parasites gained traction and studies have shown that not only do EVs play important roles in immunomodulation and parasite survival as well as disease pathogenesis in protozoan and helminth infections, they also have promising applications in the diagnosis and treatment of parasitic disease [25–27].

Over the last two decades, EVs in malaria have been investigated in human field studies as well as in in vivo, in vitro and ex vivo experimental studies. In this review, these studies and how they have advanced the understanding of the importance of host and parasite derived EVs in malaria pathogenesis and biology are examined. an analysis of the evidence supporting malaria EVs as potential biomarkers and therapeutic tools is provided. Until recently, the majority of these studies designated EVs with respect to their biogenesis as microparticles, microvesicles or exosomes without clearly demonstrating their cellular origin. Taking this into consideration, these vesicles will be referred to generically as EVs throughout this review. For studies that adopted the International Society for Extracellular Vesicles (ISEV) nomenclature guidelines with reference to size i.e., small EVs and medium EVs [28] these will be referred to accordingly.

Exosomes and microvesicles are released from viable cells. While both are membrane bound structures, exosomes originate from multivesicular bodies (MVB) and are released as vesicles of 50-100 nm in diameter from the cell following fusion of the MVB and plasma membrane; microvesicles on the other hand, are formed directly from the plasma membrane by outward budding and have a diameter of 100-350 nm though may be larger [29].

## Discovering a repertoire of multifunctional biomarkers

In parasitic infections, EVs may be released directly by the parasite, by parasite-infected host cells, and/or by effector cells in response to infection [30, 31]. Invariably, parasite infection results in elevated EV concentrations in the plasma and other body fluids [18, 30]. This was observed in early studies of human and murine malaria, all of which focused on plasma derived EVs from platelets (PEVs), endothelial cells (EEVs), red blood cells (REVs), monocytes (MEVs) and lymphocytes (LEVs).

Elevated circulating EEV levels were the first to be observed in a clinical study of Malawian children, where there was a sixfold increase in EEVs in patients with CM [32]. This was followed by a clinical study in Cameroon that sought to delineate the involvement of multiple host cells in vesiculation in malaria. From the most to the least elevated, significantly higher titres of PEVs, REVs, EEVs, MEVs, and LEVs were detected in CM patients [33]. Reports of similar EV increases were made in India, although the order of increasing cell source titres in patients with severe malaria (which included CM, non-cerebral severe malaria and multiorgan dysfunction) was PEVs, EEVs and REVs [34, 35]. A Thailand study focusing only on plasma REVs across three human malarias found them to be markedly elevated in uncomplicated falciparum malaria and most significantly elevated in patients with severe falciparum malaria [36]. This study also reported slightly higher REV concentrations in malariae malaria and vivax malaria [36], although it was previously shown in Brazil that patients with uncomplicated vivax malaria had markedly raised plasma REVs, PEVs and LEVs compared to uninfected controls [17]. In murine models of CM, platelets, endothelial cells, and red blood cells (RBCs) have been shown to be the major host cell sources of plasma EVs [37–40]. LEVs and MEVs have been the least detected [40, 41].

**Fig. 1:**
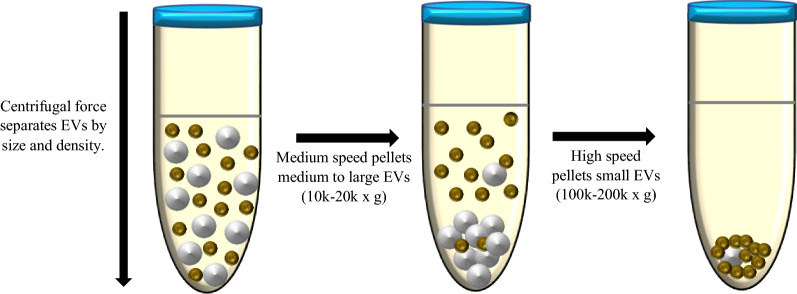
Schematic representation of EV separation by differential centrifugation. The differential centrifugation technique separates EVs according to their size and density into medium EVs (white, >200 nm) and small EVs (green, <200 nm). Medium to large EVs are first pelleted by centrifugation at 10,000–20,000×*g*. The supernatant from this initial centrifugation step is then spun down at 100,000–200,000×*g* to obtain small EVs

Arguably, there have been discrepancies in reports of the host cell sources of EVs and, therefore, discrepancies in the distribution of plasma EV populations in the different malarial infections across human populations in malaria endemic regions, as well as in murine malaria models. This observed variance, particularly in human field studies of plasma EVs in malaria [17, 32–36, 42] may be attributed to several crucial factors (Table 1). With the exception of the study conducted in Malawi [32], the sample sizes for EV quantification and classification in the clinical studies were small, ranging from 36 to 146 samples. Different EV isolation methods were employed, with some studies using medium speed centrifugation and others using high speed ultracentrifugation. These methods are sequential steps in the differential centrifugation process (Fig. 1) [43]. They separate EVs, according to their size and density, into medium EVs (mEVs, likely to be microvesicles) and small EVs (sEVs, mainly exosomes), respectively [28, 43, 44]. The majority of these studies used medium speed centrifugation, hence the few that employed ultracentrifugation may have analysed plasma EV isolates that were poorly representative of the mEV subpopulation. All these studies, however, analysed EVs using flow cytometry that is limited by low sensitivity [45] and consequently, poor inter-study comparability. Nevertheless, flow cytometry has been, and remains, an invaluable tool for EV analysis. Lastly, different host cell sources were investigated across the studies, further constraining inter-study comparability.

**Table 1:** Factors affecting inter-study comparability of human field studies of plasma EVs in malaria

*Plasmodium* species	Sample size	EV isolation method	EV analysis technique	Plasma EVs analysed	References
*P. vivax*	37	Medium speed centrifugation (14,000×*g*)	FC	LEVs, MEVs, REVs, PEVs, EEVs	[17]
*P. falciparum*	250	Medium speed centrifugation (1500×*g*)^a^	FC	EEVs	[32]
*P. falciparum*	146	Medium speed centrifugation (13,000×*g*)^a^	FC	LEVs, MEVs, REVs, PEVs, EEVs	[33]
*P. falciparum*	128	High speed ultracentrifugation (100,000×*g*)	FC	REVs, PEVs, EEVs	[34]
*P. falciparum*	128	High speed ultracentrifugation (100,000×*g*)^a^	FC	REVs, PEVs, EEVs	[35]
*P. falciparum, P. vivax, P. malariae*	36	Medium speed centrifugation (13,000×*g*)	FC	REVs	[36]
*P. falciparum*	434	Medium speed centrifugation (15,000×*g*) for mEVs; High speed ultracentrifugation (100,000×*g*) for sEVs	FC NTA TEM WBA	Total plasma EVs	[42]

*FC* flow cytometry, *NTA* Nanoparticle Tracking Analysis, *TEM* Transmission Electron Microscopy, *WBA* Western Blot Analysis; g- relative centrifugal force^a^ Indicates that the centrifugation speed was not clearly stated in the journal article

A salient observation made by the human studies was that the increased EV titres detected during acute malaria significantly decreased at the time of patient recovery, returning to baseline levels in most cases. Thus, an association between EVs and malaria was established with the consensus that EVs are potential biomarkers for disease, specifically falciparum and vivax malaria. In the case of falciparum malaria, certain published data suggested that plasma EVs may be a biomarker for CM—to be used for disease management and monitoring cure rates—since abnormally high levels of EVs correlated specifically with neurological symptoms of CM, but not other forms of severe malaria or uncomplicated falciparum malaria [32, 33]. In a recent study, however, a large cohort of patients presenting with uncomplicated falciparum malaria were shown to have markedly raised plasma mEV titres. This was considerably higher in patients under age 11 and over age 45 than in the 12–44 age group and uninfected controls [42]. As mentioned, markedly raised plasma EVs have previously been detected in uncomplicated falciparum malaria with EV levels falling drastically within 24 h after commencing anti-malarial treatment [36]. Respectively, these findings imply that plasma EVs may be useful predictors of severe disease in vulnerable age groups with poor immunity to malaria [42] and serve as pharmacodynamic biomarkers if an apparent link is made between malaria EVs and specific anti-malarial/adjunctive therapies. Furthermore, whilst these EV titres were remarkably elevated very early in infection, a positive correlation with parasitaemia was observed, suggesting parasite-infected RBCs are a major source of EVs, which would suggest that plasma EVs are potential malaria diagnostic biomarkers [42].

So far, the exception for an association between severe malaria and plasma EV levels has been placental malaria, which occurs in pregnancy. Nevertheless, EVs have been flagged as potential biomarkers for placental malaria due to overexpression of specific EV bound molecules in active placental malaria cases [46].

EVs as biomarkers for severe malaria is an enticing prospect achievable only with intensified clinical and experimental studies to substantiate their potential role. Since EVs are released under physiological and many pathological conditions, and the nature of EVs varies within and among individuals, the cargo of EVs in malaria are likely quite heterogenous and needs to be investigated thoroughly. This is imperative in determining whether or not malaria derived EVs may serve as diagnostic, monitoring, pharmacodynamic, predictive or prognostic markers. An in-depth knowledge of the diversity of EV cargo, as well as an understanding of the differences between biomarkers and their applications, will guide malaria EV research in the right direction towards validating malaria EVs as biomarkers, particularly of cerebral malaria.

Nevertheless, the assertion that plasma EVs in malaria may fulfil applications as multifunctional biomarkers has spawned several scientific questions, setting in motion a succession of functional studies centred on the pathogenic role EVs may play in severe malaria. These are presented below.

## The pathogenesis of malaria: a potential role for EVs

### Plasma extracellular vesicles

Membrane vesiculation is a cellular process that is largely dependent on the ATP-binding cassette transporter A1 (ABCA1) [47, 48]. Located primarily on the cell membrane, ABCA1 regulates the transmembrane distribution of a key lipid component of EVs, phosphatidylserine [47]. A murine model of CM showed that in ABCA1 gene knockout mice, EV shedding was drastically reduced, and the mice did not show any apparent CM-associated neurological signs or histopathology [38]. In humans, EV release was considerably lower in patients with uncomplicated malaria/ non-cerebral severe malaria and polymorphisms in the ABCA1 gene, while in patients with CM/multiorgan dysfunction and the wildtype ABCA1 gene, EV release was remarkably higher [34]. These studies show that a reduced ability of cells to vesiculate in the absence of ABCA1 or presence of “favourable” polymorphisms in this gene (in the context of malaria) confers protection against severe forms of malaria with a cerebral component, suggesting a pathogenic involvement of EVs in CM. Indeed, human polymorphisms may explain why CM occurs in less than 2 % of malaria patients [49]. ABCA1 gene is exceptionally polymorphic [50, 51] and may be at the core of the molecular mechanisms that underly the pathophysiological process by which EVs exacerbate CM.

With high plasma EV levels, there is an abundance of circulating tissue factor and phosphatidylserine (PS), both of which are procoagulant molecules that initiate coagulation and are enriched on the EV membrane [52]. In experimental CM (ECM), EVs were substantially shed in ABCA1 wild type mice. *In vitro*, these EVs had a potent procoagulant activity, the strength of which increased with increasing amounts of EVs [38]. Coagulopathy is important in the pathogenesis of CM, much of which is believed to occur in the brain [53]. EVs in CM may in fact make up a uniquely pathogenic population of EVs that preferentially accumulate in the brain and contribute to the dysregulated coagulation. It was shown that EVs, transferred from *Plasmodium berghei* infected mice with CM, localized in the brain microvasculature of recipient mice with CM but not in healthy controls or mice with non-CM [40]. This study also showed there is a rapid and acute rise in plasma EVs early in infection and during the neurological stage—both being critical timepoints in the pathogenesis of CM [40].

The cargo EVs carry is equally important as high EV titres in implicating EVs in malaria pathogenesis. Malaria EV cargo has been investigated in experimental malaria models [54–58] and human field studies [59–61] (Table 2). Proteomics identified 60 differentially abundant host proteins in ECM-EVs compared to non-infected EVs, while network analysis detected proteins implicated in fundamental pathogenic mechanisms of CM, such as endothelial and immune cell activation [57]. The presence of 2 host proteins—carbonic anhydrase 1 and S100A8—was further validated in this study. Detection of S100A8 almost exclusively in ECM-EVs, as well as two parasite proteins (intra-erythrocytic *P. berghei*-induced structures protein 1 and merozoite surface protein-1), is strongly suggestive of selective packaging into ECM-EVs [57] thereby distinguishing them from non-infected EVs and defining an association with a pathological state.

**Table 2:** Malaria plasma EV cargo from experimental malaria models and human field studies

*Plasmodium* species	Biomolecular cargo	Important findings in malaria derived EVs^a^	References
*P. yoelii*	Proteins	EVs carry reticulocyte exosome associated proteins including transferrin receptor & heat shock 70 protein No markers for microvesicles or apoptotic vesicles detected Parasite proteins with possible antigenic properties identified Parasite proteins important in host-parasite interactions identified	[54]
*P. yoelii*	Proteins	EVs carry reticulocyte exosome associated proteins including transferrin receptor & heat shock 70 protein Parasite proteins with possible antigenic properties identified Parasite proteins important in host-parasite interactions identified	[55]
*P. vivax*	Proteins	Human and parasite proteins Suitable model for identifying biomarkers of hypnozoite infection	[56]^b^
*P. berghei*	Proteins	EVs contain differentially abundant proteins important for CM pathogenesis EVs contain parasite proteins	[57]
*P. berghei* *P. yoelii*	miRNA	EVs contain significantly dysregulated miRNA species that are important for CM pathogenesis	[58]
*P. vivax*	Proteins	Parasite proteins present exclusively in infection derived EVs	[59]
*P. falciparum* *P. vivax*	miRNA	EVs contain significantly upregulated miRNAs important in malaria pathway and host-parasite interactions	[60]
*P. falciparum*	Proteins	EVs contain proteins associated with complement activation, coagulation & inflammation EVs in infection exclusively contain human RAB proteins that regulate intracellular processes and cytokine secretion EVs contain parasite proteins associated with biology & virulence	[61]

*EBA* Erythrocyte Binding Antigen, *PS* phosphatidylserine, *PI* phosphatidylinositol, *PC *phosphatidylcholine, *uREVs *uninfected RBC derived EVs, *iREVs* infected RBC derived EVs

^a^Not an exclusive summary

^b^Human liver chimeric mouse model designed to identify biomarkers of hypnozoite infection

Proteomic analysis of plasma EVs from out-patients infected with *P. falciparum* similarly identified a myriad of host proteins compared with uninfected controls [61]. Many of the identified host proteins were associated with complement activation, coagulation, and inflammation; notably 23 human RAB proteins were exclusively identified in falciparum malaria EVs [61]. RAB proteins are regulators of intracellular membrane trafficking in eukaryotic cells and modulate cytokine secretion in immune cells [62]. 29 *P. falciparum* proteins associated with parasite biology and virulence were identified, of which heat shock 70 kDa protein, Pf-enolase and Pf-actin had the highest frequencies of detection [61].

miRNAs are important regulators of gene expression. Protectively packaged into EVs, miRNAs are now known to play vital roles in protozoan and other infectious diseases [63, 64]. *Plasmodium* infection evidently alters miRNA expression, as distinct plasma EV-miRNA profiles in murine malaria models [58] and human malaria [60] have been identified. In EVs from *P. berghei* ECM, the expression of miR-146a and miR-193b was found to be significantly dysregulated compared to EVs in *Plasmodium yoelii* non-CM and non-infected mice; while miR-146a was markedly upregulated in EVs, miR-193b was downregulated [58]. In humans, when compared with uninfected individuals, the miRNA hsa-let-7a-5p was upregulated in EVs from patients with falciparum and vivax malaria, while miRNAs hsa-miR-150-5p and hsa-miR-15b-5p were both upregulated in vivax malaria alone [60]. These and other miRNAs have already been implicated in other neurological diseases and were predicted to be of importance in pathways relevant to malaria, such as EV biogenesis, inflammation, cytoadhesion, [58], adherens junction and transforming growth factor (TGF)-β pathways [60]. Furthermore, hsa-let-7a-5p miRNA, which was found in EVs from falciparum and vivax malaria patients, has known roles in host-parasite interactions [60].

Although the role of miRNAs in CM pathogenesis is unknown, the notable degree of altered expression in EVs from mice with CM and human malarial infection, as well as preliminary data from in vitro EV experiments on *Plasmodium* (discussed below), suggest a regulatory role for miRNAs in this disease. Moreover, miRNAs have been found to be differentially expressed in the brains of mice with CM and non-CM [65, 66]. In one study, miR-155, which alters the integrity of the blood brain barrier (BBB), was increased in plasma EVs isolated from CM susceptible mice relative to resistant mice. Following miR-155 knockout in these susceptible mice, survival was significantly improved and there was less damage to the BBB [67]. In a field study of pregnant women, miR-517c was twice as likely to be detected in EVs from women with active placental malaria than in uninfected pregnant women [46].

Focusing on *P. falciparum* malaria either in natural human infections or through extrapolations from murine CM studies, the data from gene knockouts, omics studies and gene association have cumulatively inferred a potential role for plasma derived EVs in malaria. Importantly, a study using *P. vivax* has rendered the first evidence of a direct pathophysiological mechanism by which plasma EVs in natural malaria infection may contribute to the pathogenesis of this disease [59].

It was noted that plasma EVs from acute human *P. vivax* infection are comprised mainly of a CD71^+^ EV (<200 nm) sub-population that carries human and parasite proteins, cautiously suggesting infected reticulocytes might be the main source of EVs in *P. vivax* infection [59]. Based on their observations, the authors described a mechanism where *P. vivax* infection derived EVs are actively taken up by human spleen fibroblasts, followed by translocation of the NF-kB transcription factor from the cytoplasm to the nucleus. This induces a TNF-independent upregulated surface expression of ICAM-1 on spleen fibroblasts, which results in the adherence of mature schizont stage *P. vivax*-infected reticulocytes to these cells [59]. The active uptake of plasma derived *P. vivax* EVs by spleen cells had been previously demonstrated [55]. This EV/ NF-kB/ ICAM-1 cytoadherence pathway is a novel discovery that merits validation and further investigation to uncover plasmodial molecules that may be directly involved and expose details of the mechanism. Since *P. falciparum* and *P. berghei* can infect reticulocytes, the discovery of this EV-directed pathway in CM would be quite compelling and stimulate new research foci.

Only recently has the potential severity and lethality of vivax malaria been acknowledged [11], the pathogenesis of which remains largely obscure. Cytoadherence and sequestration have been proposed as pathogenic mechanisms that *P. vivax* may have in common with *P. falciparum* [68, 69]. As laid out in the study by Toda and colleagues [59], *P. vivax* derived EVs may have a crucial role to play and set forward a paradigm shift in the understanding of *P. vivax* biology, its interaction with the human host and pathogenesis. Indeed, previously unknown aspects of *P. falciparum* biology and its communication with host cells have come to light in *in vitro* EV studies and will be discussed in later sections.

### Cell-specific extracellular vesicles

The biomolecular constitution of EVs and their parent cells are as similar as they are different. This paradox is testament to a host of EV features, including (1) selective EV cargo packaging, (2) differential biodistribution and target cell-specificity, (3) distinct downstream cellular signalling pathways activated by EVs, and (4) specialized functions different from their cells of origin [23, 70–72]. EVs from different cells perform some common functions but certain EV populations primarily fulfill specific physiological and pathological roles, such as coagulation, inflammation, angiogenesis, cell cytotoxicity, antigen presentation, and immunoregulation. This phenomenon has been extensively reviewed [23, 73–75]. As such, the extent to which cell specific EVs contribute to different disease processes varies, and this has implications for their application in the diagnosis and treatment of diseases [76].

While the scientific evidence for a potential role of EVs in malaria gathered from clinical and experimental cerebral malaria studies is compelling, they are generalizations of plasma EVs from humans and mice. Over the last 2 decades, there have been a number of studies on cell specific EVs derived from the vascular endothelium, platelets, and RBCs. While these studies have generated more questions than answers, they have offered new insights into EV characteristics, and the potential involvement of EVs in the pathology of malaria. Importantly, a critical evaluation of the findings from these studies will direct future research.

#### Endothelial cell derived EVs (EEVs)

Endothelial cell (EC) activation is a core component of the pathophysiological events that occur in falciparum malaria. This is consequent of the distinctive cytoadherence of mature-stage infected RBCs (iRBCs) to the microvascular endothelium of various tissues and organs, including the brain in CM [12, 77]. ECs are known to respond to various stimuli [75]. One such response, demonstrated *in vitro*, is the shedding of EVs from TNF-activated ECs in thrombotic disorders [78]. Coupled with the knowledge of high TNF levels in malaria [79, 80], this observation prompted the first study of endothelial cell-derived EVs in this disease [32].

Patient derived-ECs in CM and non-CM have been shown to respond differently to TNF stimulation *ex vivo* with significantly increased shedding of EVs in the former [81]. Considering the important role TNF plays in the pathology of CM (15), differential EEV shedding in response to this pro-inflammatory cytokine is substantial enough to warrant further investigation of the role EEVs may play in this disease. For the first time, in 2014, a series of experiments investigated the pathogenicity of TNF-activated EEVs in mice [40]. When *in vitro* generated EEVs were injected into healthy mice, they were rapidly cleared from circulation and induced lesions in the brain and lungs, similar to those seen in human CM (HCM) and ECM [40]. These findings suggest that the brain and lungs may be preferred EEV sites where they cause cell damage, and provides evidence for their potential pathogenicity in CM.

The correlation between EEVs and TNF, particularly in HCM [32, 35, 81], as well as the consideration that EEV shedding is a secondary pathologic effect of TNF production, were the rationales behind a study of the anti-inflammatory molecule LMP-420 to indirectly inhibit EV production [82]. LMP-420 is a potent inhibitor of TNF synthesis. It was found that, when this molecule was added simultaneously with TNF to human brain microvascular ECs, EV release from these cells was reduced by up to 50 % compared to ECs treated with TNF alone [82]. Although this study was performed in the context of CM, the hypothesis that blocking EEV release would prevent the development of ECM or HCM was not tested in vivo. The significant reduction of EV release from TNF-activated ECs was observed in another *in vitro* inhibition study, where ECs were treated with pantethine before TNF activation [83]. Pantethine is a thiol provitamin known to directly prevent EV shedding by impeding phosphatidylserine translocation to the outer cell membrane leaflet, a process required for EV shedding [84]. In vivo experiments showed that mice treated with pantethine 24 h post-infection with *P. berghei* did not display any signs of CM, which was attributed to two crucial events: diminished platelet reactivity, and downregulated EV production [83]. Another compound, citicoline, was found to reduce EV production in human brain ECs in vitro, and significantly enhance survival of mice with CM when used in combination with artesunate [85]. Citicoline is a widely available dietary supplement that occurs naturally in eukaryotic cell membranes and plays a role in membrane stability. The prospect of the availability of EEV-inhibitory drugs as adjunctive therapies for the treatment or prevention of CM is encouraging yet improbable without an in-depth understanding of the pathogenic mechanisms of EEVs in malaria. Though complex, an experimental model with ex vivo, in vitro, and in vivo components may be essential to provide insights into a direct role of malaria induced EEVs in the pathogenesis of this neurological syndrome.

To adequately investigate the function of EEVs, specifically in plasma, they must be properly characterized in the midst of EVs from other cell sources. However, EEV characterization is problematic and poses a challenge for downstream EEV studies [75]. In the clinical study in Malawi, markedly elevated circulating plasma EEV levels in paediatric CM patients was the first indication that EVs may be a biomarker for malaria [32] (see above). However, EEVs as a predictor of fatality is uncertain due to conflicting data published on the EEV subpopulations. In Malawi, CD51:EEVs were characterized, and lower levels were found at the time of hospital admission in CM patients who died compared to those who survived [32]. In Cameroon, CD51:EEVs and CD105:EEVs were characterized. Higher titres of CD105:EEVs were recorded overall, as well as significantly higher titres at the time of hospital admission in patients who died compared to those who survived [33]. These are only 2 examples of the numerous EEV phenotypes, as parent endothelial cells carry multiple surface markers, including CD31, CD54, CD62E, CD106, CD144 and CD146 [81, 86]. While only CD144 and CD62E are exclusively expressed on ECs, activated monocytes/macrophages also express CD105 and CD51 [86]. Therefore, analysis of a combination of EEV markers is necessary to minimize ambiguity when assessing the origin and role of EEVs in malaria. A study of a murine model of ECM, in which CD144:EEVs were characterized, failed to show a significant difference in EEV titres between malaria-infected and uninfected mice, and the authors concluded that endothelial cells are not an important source of EVs in CM [39]. This data echoes earlier reports from similarly designed ECM models (also using CD144) that showed ECs account for less than a third of total plasma EVs [38]. However, it should be noted that CD144 is not an ideal marker for detecting EEVs [86]. As seen with other diseases [75, 86], in order to obtain reliable data on malaria induced EEVs, it is essential that future studies use multiple EC surface markers to improve sensitivity and specificity of detection.

#### Platelet derived EVs (PEVs)

Platelets, like endothelial cells, play an important role in the pathogenesis of CM. Studies have shown platelets to be involved both functionally (such as by releasing potent inflammatory cytokines) and physically (by accumulating in the brain and directly promoting iRBC cytoadherence) reviewed in [19, 41]. Abnormally low levels of platelets are known to occur in ECM [87] and HCM [88]. CM-associated thrombocytopenia remains poorly understood, nevertheless, through a series of in vitro and in vivo studies, it is known to involve caspase-dependent disintegration of platelets that results in the excessive shedding of PEVs in the plasma of *P. berghei*-infected mice [89]. Correspondingly, in humans, PEV levels have been found to be specifically and markedly elevated in thrombocytopenic patients presenting with CM [33]. Piguet and colleagues observed that treatment with the caspase-inhibitor, Z-Val-Ala-Dl-Asp-fluoro-methylketone (ZVAD-fmk) significantly reversed the inverse correlation between PEVs and platelets in the plasma of infected mice and increased the survival of the animals [89]. It is unknown whether survival in these mice was directly linked to the reduction in plasma PEVs. However, in HCM, plasma PEV levels, originally elevated in patients with acute disease, declined considerably at the time of their hospital discharge [33].

A clear relevance for PEVs has been described in various pathologic conditions, including those caused by infectious diseases [18]. In experimental and human CM, studies have shown that platelets account for more than 50 % of total plasma EVs [33, 38, 89]. Moreover, since platelets can bridge interactions between endothelial cells and iRBCs by providing the necessary surface adhesion molecules [90] that are also present and enriched on the surface of PEVs [18, 72], it was postulated that elevated circulating PEVs was an indication of their potential role in the pathogenesis of CM. This postulation was eloquently investigated in an *in vitro* model of HCM by Faille and colleagues [91]. Among their findings were that, in CM (1) in a dose-dependent fashion, PEVs preferentially bind to iRBCs rather than to uninfected RBCs, (2) binding between PEVs and iRBCs directly involves multiple platelet adhesion molecules (including PECAM-1 & GPIV) and *P. falciparum* erythrocyte membrane protein 1—PfEMP1, (3) PEVs adhere to the surface of iRBCs and human brain ECs but only translocate across the membrane of the latter, (4) PEVs can transfer PECAM-1 & GPIV to the surface of human brain ECs, and (5) iRBCs are more adherent to ECs in the presence of PEVs [91]. The results of this study provide an understanding of the potential role of PEVs in cell-cell interactions, cytoadherence and RBC sequestration in CM.

These findings have not been directly corroborated in vivo. However, in a clinical study in Cameroon, elevated PEV levels in children correlated with the severity of coma [33]. It may be argued that the correlation between PEV levels and coma severity observed in this study was due to increased sequestration of iRBCs in the brain microvasculature. This is in accordance with the ability of PEVs to increase iRBC cytoadherence to brain ECs, as observed *in vitro* by Faille and colleagues. Sequestration in vital organs other than the brain is an important element of the pathological picture of CM [92] and proadhesive PEVs may contribute to iRBC cytoadherence and sequestration in multiple organs. A property of PEVs that has been observed in physiological conditions [93] and under high shear stress [94] is the enhancement of thrombus formation at a substantially greater capacity than platelets. In CM, cerebrovascular thrombus formation is a pathological feature that commonly presents alongside cerebral sequestration [95, 96]. Thus, PEVs may be new players in CM vascular pathology. Indeed, the proadhesive and procoagulant properties of PEVs, and their potential involvement in CM pathogenesis, require critical investigation.

#### Red blood cell derived EVs (REVs)

RBCs accommodate *Plasmodium* spp. for the most part of their life cycle in the vertebrate host during which the parasite extensively modifies the structure and function of the erythrocyte. *Plasmodium* spp. synthesize and export numerous proteins that are presented on the RBC surface membrane [97]. Systemic inflammation is characteristic of CM and results when brain vascular endothelial cells and immune cells recognize parasite proteins on the surface of iRBCs or from ruptured iRBCs [14, 15, 49]. Like many other eukaryotic cells, mature RBCs constitutively shed EVs, more precisely microvesicles [98, 99]. Since microvesicles directly bud off plasma membranes, they may carry membrane-bound plasmodial molecules in malaria infection with immunogenic properties. EVs derived from *Plasmodium*-infected RBCs have been found to induce systemic inflammation in ECM [39].

Couper and colleagues performed studies on *P. berghei*-infected RBC derived EVs (pb-iREVs) where they found that pb-iREVs carried distinct parasite cargo and activated macrophages to release TNF *in vitro*, specifically through a TLR-4/MyD88 dependent pathway [39]. These pb-iREVs were much more potent activators of macrophages than viable *P. berghei*-infected RBCs, and the most immunogenic pb-iREVs were detected at the onset of clinical signs, which, in turn, correlated with peak pb-iREV levels. The authors of this study concluded that an accumulation of pb-iREVs with a distinct pro-inflammatory phenotype contribute to the development of severe malaria [39]. In-vitro studies of *P. falciparum* demonstrated the active uptake of *P. falciparum*-infected RBC derived EVs (pf-iREVs) by monocyte-derived macrophages. The pf-iREVs not only had a potent proinflammatory effect on macrophages, they also induced the expression of anti-inflammatory cytokines [100]. Mantel and colleagues showed that, while pf-iREVs are also capable of activating neutrophils, human monocytes are the main target immune cells [100].

Demonstrating with monocytes, iREVs have been implicated as central players in a novel mechanism of immune cell activation by plasmodial molecules. Nucleic acid-containing pf-iREVs are actively taken up by monocytes [101, 102]. Within monocytes, endogenous *P. falciparum* DNA cargo from these EVs is released into the cytosol, where it induces the secretion of type 1 interferons (IFN) and other cytokines in the cell via STING (Stimulator of Interferon Genes) activation [102]. STING is a cytosolic adapter protein that is activated by DNA-binding proteins and is essential for the production of type 1 IFN in innate immune cells [103, 104]. The mechanism described by Sisquella and colleagues was verified using imaging studies that clearly demonstrated the nuclear translocation of the activated transcription factor - interferon regulatory factor 3 (IRF3) in monocytes transfected with *P. falciparum* DNA [101]. IRF3 translocation is the final step in the STING-dependent signalling cascade before transcription of type 1 IFN genes [105]. How plasmodial DNA accesses the cytosolic milieu of immune cells to induce the STING-dependent innate immune response [106, 107] is poorly understood. Delivery of the DNA via haemozoin has been proposed [107], although there is controversy regarding haemozoin-DNA binding [108]. DNA delivery by iREVs offers an alternative explanation, in which case, the DNA is protected within an enclosed vesicle. Nevertheless, the role of EVs in presenting parasite DNA to immune cells in malaria requires a lot more research to answer important questions such as the mechanisms of nucleic acid loading into EVs, EV uptake by immune cells, and release of DNA cargo into the cytosol. Moreover, in depth investigation of the EV-DNA dependent stimulation of immune cells would provide insights into the pathogenesis of CM and the role of type 1 IFNs, since it has been shown that a disruption of the STING cascade significantly increases survival in knock-out *P. berghei* infected mice that are otherwise susceptible to CM [107].

There is evidence from a recent in vitro study suggesting that parasite derived EVs are more directly involved in the immunopathogenesis of CM. pf-iREVs activated human monocyte-derived microglia, resulting in an upregulation of TNF-α and simultaneous downregulation of IL-10 [109]. Microglia are the resident macrophages of the brain. Hence, this finding that pf-iREVs induce an immunomodulatory response in microglia is in line with that of Mantel and colleagues [100]. An imbalance in the inflammatory response that tips the scale toward excessive production of pro-inflammatory cytokines constitutes neuroinflammation [15]. Also characteristic of neuroinflammation is the activation of astrocytes, which are non-neuronal cells with core homeostatic functions in the brain [110]. Interestingly, pb-iREVs were rapidly taken up by astrocytes compared to iRBCs and, although these EVs were not directly implicated in activation of astrocytes, high levels of TNF were detected in co-cultures of astrocytes and *P. berghei*-iRBCs that likely contain iREVs [111]. Furthermore, in vitro studies showed that pf-iREVs induced pro-inflammatory cytokine release and VCAM1 expression in human ECs, but the most compulsive discovery was that they contain host-derived RNA-induced silencing complexes (Ago2-miRNA) that directly disrupt endothelial barrier functions [112].

Altogether, these findings implicate iREVs as facilitators of immunopathology and vascular dysfunction in malaria by virtue of the biomolecular cargo they carry. Several *P. falciparum *in vitro studies have comprehensively analysed the protein [100, 113, 114], nucleic acid [100, 102, 112, 115, 116], and lipid content of iREVs [117, 118] (Table 3). Independent proteomic analyses of EVs from rodent *P. yoelii* infections [54, 55] and plasma derived EVs from *P. vivax* infections of liver-chimeric humanized mice [56] have also been performed (Table 2).

**Table 3:** Malaria EV cargo derived from EVs of in vitro (cell cultured) iRBCs

*Plasmodium* species	Biomolecular cargo	Important findings in malaria derived EVs^a^	References
*P. falciparum*	Proteins, nucleic acids (small RNA, mRNA)	Abundant RBC lipid raft proteins Abundant resident Maurer’s cleft parasite proteins Parasite proteins involved in RBC invasion Abundant immunogenic EBA on EV surface EV content is conserved across *P. falciparum* strains miRNA species may be involved in gametocytogenesis	[100]
*P. falciparum*	Nucleic acids (RNA & DNA)	EVs contain human & parasite small RNA HmiR-451a is the most abundant specie Majority of detected miRNA regulate cell adhesion Ring stage iREVs contain plasmodial gDNA iREV-plasmodial gDNA triggers an innate immune response	[102]
*P. falciparum*	Nucleic acids (miRNA)	hmiR-451a species is present in abundance hmiR-451a is functional and complexed with hAgo-2	[112]
*P. falciparum*	Proteins	RBC proteins include haemoglobin and those associated with the membrane Significant enrichment of resident Maurer’s cleft parasite proteins and other “virulence associated proteins” Important knob-associated parasite proteins also found	[113]
*P. falciparum*	Proteins	PfEMP1 in ring stage iREVs	[114]
*P. falciparum*	Nucleic acids (miRNA)	Human miR-451, miR-486, miR-181a identified in iREVs & uREVs Significantly higher hmiR-451 levels in iREVs than uREVs Human miRNAs are complexed with Ago-2	[115]
*P. falciparum*	Nucleic acid (small RNA)	>90 % of iREV small RNA content is human Human miRNAs, tRNAs, Y-RNAs, vault RNAs, snoRNAs and piRNAs present HmiR-451a is the most abundant Host & plasmodial small RNAs involved in drug resistance detected	[116]
*P. falciparum*	Lipids	Enrichment of PS & PI Enrichment of sphingolipids with signaling functions important in immunomodulation	[117]
*P. falciparum*	Lipids	No significant difference between the lipid profile of iREVs and uREVs Enrichment of PC	[118]

*EBA* Erythrocyte Binding Antigen, *PS* phosphatidylserine, *PI* phosphatidylinositol, *PC *phosphatidylcholine, *uREVs *uninfected RBC derived EVs, *iREVs * infected RBC derived EVs

^a^Not an exclusive summary

^b^Human liver chimeric mouse model designed to identify biomarkers of hypnozoite infection

## The biology of malaria: novel features from an EV perspective

The immunopathobiology of malaria is primarily attributed to variant surface antigens (VSAs) that mediate ‘direct’ cell-cell interactions between parasite-infected RBCs and other host cells [13]. VSAs are the most studied feature of plasmodial virulence and biology [119] but much remains to be discerned about the complex nature of *Plasmodium* spp. and host-parasite interactions. Suffice to say, the growing malaria EV research over the last twenty years answers a call to fill in knowledge gaps by broadening the scope of investigation to other important components of malaria pathobiology. In various protozoan infections, parasite-derived or parasite-induced EVs are known to mediate ‘indirect’, long distance host-parasite and parasite-parasite interactions to promote virulence, immune evasion, survival, and transmission of invading pathogens, reviewed in [26, 120].

Using co-cultures of diverse parasite strains, Regev-Rudzki and colleagues demonstrated that ring stage pf-iREVs transfer episomal drug resistance genes from resistant to susceptible *P. falciparum* strains to enhance survival and gametocytogenesis under drug pressure [121]. This increase in sexual differentiation following exposure of asexual blood stage parasites to iREVs was also observed by another group, although not in the presence of drugs [100]. Rather, Mantel and colleagues observed that the rate of gametocytogenesis increased with increasing concentrations of iREVs in parasite cultures, implying this to be an inherent attribute. The favored uptake of iREVs by other iRBCs relative to uninfected RBCs [100] and the presence of regulatory microRNAs and plasmodial DNA in these EVs (see Table 3) substantiate the probability of a unique pathway by which *Plasmodium* spp. propagate their life cycle in natural infections. The implication of this pathway, should it occur in humans infected with malaria, is the potential of tackling drug resistance via novel strategies and developing drugs and vaccines that target iREVs to block the development of transmission stages of the parasite [121].

Contrary to increased sexual differentiation under drug pressure and a resultant low or controlled parasitaemia, *P. falciparum* responds to high density stress in vitro by releasing a subpopulation of EVs with differentially abundant parasite lactate dehydrogenase (LDH) that, in recipient parasites, induces apoptosis, thereby regulating parasite population [122]. Programmed cell death can be induced in human malarial infections [123] and evidence of EV-mediated PfLDH suicide signalling in vivo would have implications for antimalarial drug development.

Other iREV-bound parasite proteins—histidine rich factor (HRF), elongation factor 1α (Ef-1α) [124] and PfEMP1 [114]—have been implicated in immune evasion. When monocytes were treated with PfEMP1-carrying iREVs, genes involved in response to stress and cytokine stimulus, as well as response to defence, were not upregulated, whereas following treatment with PfEMP1-deficient iREVs, monocytes showed a significant upregulation of these genes [114]. In *P. berghei* rodent infection, compared to iREVs from HRF and Ef-1α knock-out mice, wild type sourced iREVs efficiently inhibited T cell proliferation in vitro and in vivo [118]. Over 3 decades ago, malaria antigens were reported to evade immune responses by inducing humoral and cell-mediated immunosuppression directly, albeit through poorly understood mechanisms [125, 126]. The work by Demarta-Gatsi et al. and Sampaio et al. paved the way in elucidating such mechanisms, as these studies suggest that iREVs are specifically loaded with virulent proteins that, upon delivery to immune cells, suppress the cells response to the parasite. Many more parasite proteins have been detected in iREVs [100, 113, 114, 124] and may work independently or in concert to suppress immune cell responses.

The presence of parasite biomolecules in iREVs is apparently purposeful and it has been proposed that malaria parasites direct cargo-specific loading of iREVs to exert effects in target cells for their benefit [102, 116]. This is likely, considering the extent to which malaria parasites modify the red blood cell [97]. However, neither the mechanism by which biomolecules are packaged into iREVs, nor the biogenesis of these EVs are known. Regev-Rudzki and colleagues have identified a protein - PfEMP1 trafficking protein 2 (PfPTP2) - that may be directly involved in iREV biogenesis. PfPTP2 functions in intercellular communication and preliminary data cautiously suggests that iREVs are derived from PfPTP2-coated vesicles that bud off Maurer’s clefts [121]. Mantel and colleagues agree that Maurer’s clefts are a central part of iREV biogenesis but argue, based on proteomics and live imaging data, that they are formed by blebbing directly from the RBC membrane [100]. In either case, involvement of the malaria parasites molecular and biological machinery cannot be excluded, especially since iREVs are produced from RBCs harbouring all parasite life stages [36].

As the malaria parasite matures within the RBC from ring stages, through trophozoites to schizonts, iREV production increases steadily [36, 100]. While schizont stages of *P. falciparum* may signal peak EV release from infected RBCs [100], it is the ring stages that signal specific loading of virulence factor PfEMP1 into EVs [114]. Moreover, the general distribution of proteins in EVs from iRBCs differs through the parasite’s development stages, implying a parasite driven mechanism [114] exists to generate subpopulations of effector EVs that promote stage-specific survival via different downstream signalling pathways in different host cells.

## Malaria prevention and control: the influence of EVs in therapeutics and vaccine development

Vector control and targeted chemoprophylaxis are the main malaria prevention and control strategies [2]. While widespread drug resistance in malaria poses a major challenge for successful chemoprophylaxis and treatment, development of an effective vaccine has been hampered by antigenic variation and the overall complex life cycle of *Plasmodium* spp. Studies of iREVs have identified novel malaria pathways with potential drug targets and anti-malarial host-derived EV cargo, as well as EV-associated parasite molecules already under evaluation in vaccine trials. In fact, a few studies have demonstrated the potential of whole malaria derived EVs as vaccines.

In an experimental model of malaria, mice vaccinated with CpG-ODN adjuvanted EVs derived from non-lethal *P. yoelii*-infected reticulocytes mounted a parasite-specific humoral immune response [54] accompanied by the induction of effector memory T cells in the spleen [55]. Ultimately, these mice were immunized and protected from primary and subsequent lethal *P. yoelii* infections [54, 55]. *Plasmodium yoelii*-infected reticulocyte-derived EVs revealed over 70 *Plasmodium* proteins, including the immunogenic rhoptry proteins and merozoite surface protein 1 (MSP-1) [55]. Together, these studies suggest an antigen-presenting role for malaria-derived EVs and opened up a new avenue for malaria vaccine development [54, 55]. Similarly, in the recent study by Demarta-Gatsi and colleagues, malaria-naïve mice inoculated with iREVs from *P. berghei* infected mice survived an initial lethal infection and developed long-lasting immune memory that conferred protection against a second infection [124]. The authors noted a genetic component may have also determined survivability, since survival rates varied in the different mouse strains tested. EF-1α was identified as an immunogenic EV-associated protein. Mice immunized with recombinant EF‐1α also survived infection, however, parasite clearance required a longer period of time in these mice than in those immunized with EF‐1α-containing iREVs [124]. Although not clearly noted in the study, this likely implies a higher immunogenicity of iREVs and their suitability for controlled and targeted delivery of immunogens to induce a favourable immune response. Combined with biocompatibility and stability, these features are why the use of EVs as cancer vaccines and in drug delivery is gaining popularity over synthetic vesicle delivery systems [127, 128].

A number of earlier malaria vaccine studies focused on developing synthetic nanoparticles and microparticles for the delivery of parasite genes or proteins and a majority of the formulations included the biocompatible polymer poly (lactic-co-glycolic acid)—PLGA [129–134]. For the first time, iREVs from in vitro *P. falciparum* cultures and corresponding uninfected RBC derived EVs (uREVs), have been evaluated for their potential as natural antimalarial drug delivery systems [118]. Two lipophilic antimalarial drugs—atovaquone and tafenoquine—when loaded into iREVs and uREVs via simple co-incubation, inhibited the growth of *P. falciparum *in vitro more efficiently than when the parasites were exposed to free unencapsulated drugs [118].

Amongst other advantages of natural EVs, iREVs were bound by iRBCs and uRBCs with significantly greater avidity than uREVs, making this novel targeted drug delivery system a promising approach to improving antimalarial therapy and tackling drug resistance [118]. It also holds strong promise for the development of adjunctive immunotherapies for CM, since various studies have already shown that iREVs are actively taken up by endothelial cells [112], monocytes [100, 102, 114], glial cells [109, 111], T cells [124] and natural killer cells [135].

Uptake of iREVs by natural killer (NK) cells is particularly appealing for anti-malarial therapy and deserves thorough investigation. When pf-iREVs, NK cells and iRBCs were co-cultured, iREVs (in a dose-dependent fashion) altered the phenotype of a ‘non-responsive’ population of NK cells to a ‘responsive’ phenotype with concomitant reduction in parasitaemia [135]. NK cells are crucial for a protective innate immune response against blood stage parasites, as they are a primary source of IFN-γ required for controlling parasitaemia [136], and severe malaria in patients has been associated with a preponderance of ‘non-responding’ NK cells [135]. iREVs were implicated in stimulating NK cell activity via their RNA content. iREV associated RNA primed NK cells by activating MDA5 (melanoma differentiation associated gene 5), as indicated by significant upregulation of CD69 activation marker expression but poor stimulation of IFN-γ production [135]. MDA5 is a sensor for cytosolic foreign RNA, the activation of which triggers an innate immune response [137] crucial for determining the outcome of malaria infection [135]. Based on their observations, Ye and colleagues proposed that plasmodial RNA in EVs was directly released into the cytosol following fusion of the EV and NK cell membranes [135]. Direct contact between iRBCs and NK cells is a precondition for optimal IFN-γ production against *Plasmodium* [138] and data from this study further suggested that iREVs may activate NK cells indirectly by enhancing direct cell-cell contact between iRBCs and NK cells through the induction of high-affinity lymphocyte function-associated antigen 1 (LFA-1) [135, 139]. Identifying the RNA species in iREVs responsible for priming/activating NK cells would suggest natural EVs carrying specific functional RNA could be developed [127] as an NK cell-based antimalarial treatment [135].

On the other hand, functional human miR-451 and miR-140 complexes in EVs from uRBCs have been shown to negatively impact *P. falciparum* virulence [115]. Malaria infection signalled the release of EVs from uRBCs. When uREVs were taken up by iRBCs, EV-associated hmiR-451 and hmiR-140 downregulated the expression of genes encoding the virulence protein PfEMP1. Furthermore, iREVs, as well as uREVs, inhibited RBC invasion by merozoites [115]. The unfavourable effect of uREV hmiRNA complexes on parasite survival [115] contrasts the pathophysiologic effect of iREV hmiRNA complexes on host endothelial cells [112] mentioned earlier. This reiterates the complexities of malaria infection and the need for comprehensive studies, preferably using clinical *P. falciparum* isolates [113, 140] and rodent malaria parasites [141] to gain insights into the dynamic interaction between iREVs, uREVs and the vascular endothelium and the factors that may determine their pathogenic or protective effects in vivo. In the meantime, by virtue of their ability to hinder parasite survivability and control parasitaemia, host RBC-derived miRNAs hold promise as a novel malaria drug [115] that can also be delivered in EVs.

Malaria-derived EVs may hold potential beyond antimalarial therapeutics. In a murine model of cancer, intriguing pathological and immunohistochemical data showed the tumor-suppressive and anti-angiogenic properties of plasma derived EVs from *P. yoelii*-infected mice [142]. The authors concluded that certain miRNA species were enriched in plasma EVs consequent of *P. yoelii* infection and these miRNAs directly inhibited expression of an essential regulatory gene for growth and migration of vascular endothelial cells [142].

## Conclusion

The EV network in malaria is complex, with extensive crosstalk at a molecular and cellular level among numerous host cells, within parasite populations, and between host cells and parasites. The correlation between circulating EVs and disease severity in natural infections, as well as protection against ECM by gene knockouts and pharmacological inhibition, supports the potential for EVs as biomarkers and suggests a pathogenic role in disease. A protective function of some EV populations has also been proposed. The many attributes of EVs are bestowed by their biomolecular cargo. Although the understanding of malaria EV biology is gradually advancing, there remains significant gaps in the knowledge of the distinct nature of EVs released by the different life stages of malaria parasites as well as how this may vary from one individual to another. More so, the mechanisms of specialised cargo loading, EV release and EV uptake are yet to be elucidated. This is essential for deeper understanding of the pathogenic and protective pathways of EVs, which in turn is necessary for the successful application of EVs as therapeutics and vaccines to intercept or promote these pathways. Furthermore, the notion that EVs may be used in the development of malaria vaccines requires substantially more scientific evidence. Technologies need to be developed that allow the manufacturing of homogenous EV populations and the purification of EVs from malaria cultures in large enough scale for vaccine production.

Nevertheless, as for understanding the involvement of cell specific EVs in malaria pathogenesis, host-parasite interactions, and the complex biology of *P. falciparum* and *P. vivax*, novel details have emerged from numerous studies over the last 2 decades. Much remains to be learned, and the field of malaria will likely see more research focusing on the characterization of distinct malaria EV populations, their biogenesis and fate, and critically investigating their role in disease.

## Data Availability

Not applicable.

## Abbreviations

ABCA1: ATP-binding cassette transporter A1
BBB: Blood brain barrier
CM: Cerebral malaria
EC: Endothelial cells
ECM: Experimental cerebral malaria
EEVs: Endothelial cell-derived extracellular vesicles
Ef-1α: Elongation factor 1α
EVs: Extracellular vesicles
HCM: Human cerebral malaria
HRF: Histidine rich factor
iRBCs: Infected red blood cells
iREV: Infected red blood cell-derived extracellular vesicles
IRF3: Interferon regulatory factor 3
ISEV: International Society for Extracellular Vesicles
LDH: Lactate dehydrogenase
LEVs: Lymphocyte-derived extracellular vesicles
mEVs: Medium extracellular vesicles
MEVs: Monocyte-derived extracellular vesicles
MDA5: Melanoma differentiation associated gene 5
MVB: Multivesicular bodies
NK: Natural Killer cells
pb-iREVs: *P. berghei*-Infected RBC derived EVs
PEVs: Platelet-derived extracellular vesicles
PfEMP1: *P. falciparum* Erythrocyte membrane protein 1
PfPTP2: PfEMP1 trafficking protein 2
pf-iREVs: *P. falciparum*-Infected RBC derived EVs
PS: Phosphatidylserine
RBCs: Red blood cells
REVs: Red blood cell-derived extracellular vesicles
sEVs: Small extracellular vesicles
STING: Stimulator of Interferon Genes
TNF: Tumor necrosis factor
uREVs: Uninfected red blood cell-derived EVs
